# The impact of war on Primary Health Care in Ukraine: a cross-sectional survey and qualitative interviews with service providers

**DOI:** 10.1186/s12913-026-14555-6

**Published:** 2026-05-09

**Authors:** Adrianna Murphy, Kaija Kasekamp, Olga Demeshko, Jarno Habicht

**Affiliations:** 1 London School of Hygiene and Tropical Medicine, Keppel Street, London, WC1E 7HT UK; 2 WHO Barcelona Office for Health Systems Financing, Sant Pau Art Nouveau Site (La Mercè Pavilion), Sant Antoni Maria Claret 167, Barcelona, 08025 Spain; 3 World Health Organization Country Office in Ukraine, Mykhaila Hrushevskoho St, 9B, Kyiv, Ukraine

**Keywords:** Primary Health Care, Conflict, Ukraine, Health care financing

## Abstract

**Background:**

Primary Health Care (PHC) is vital to supporting emergency preparedness and health care resilience. There is limited evidence of the impact of crises on PHC services and financing. We aimed to explore the impact of the full-scale invasion of Ukraine in February 2022 on PHC services in the country.

**Methods:**

We used a mixed-methods approach. Survey data were collected using an online questionnaire sent to a sample (*n* = 86) of PHC providers in Ukraine in January-February 2023. Fifteen providers were then randomly selected for semi-structured interviews from among those that reported an impact of war and from those areas most affected by conflict. Interviews took place in March 2023.

**Results:**

37% of PHC providers reported being affected by the full-scale invasion. Qualitative data revealed greater impacts at the beginning of the invasion, to which facilities adapted by the time of the survey. The most reported disruptions were electricity cuts (76%) and currency depreciation/price increases (72%). The most reported increased medical need was cardiovascular disease (CVD; 58%) (with qualitative data suggesting an increase in CVD among younger patients) followed by mental illnesses and disorders (55%). 59% of PHC providers reported an increase in remote consultations. Among those facilities that reported a change in revenues, the nature of the change depended on the type of ownership. For example, only 9% of private providers reported increased revenues from humanitarian aid, while 79% (*n* = 58) of public providers indicated an increase in these sources.

**Conclusion:**

To continue strengthening Ukraine’s PHC system, the benefit package must be aligned with clinical guidelines, particularly for CVD and mental health; increases in remote consultations should be closely monitored for quality; and payment systems must be adjusted to ensure equity of financing regardless of provider ownership. These findings offer insights for strengthening PHC and emergency-preparedness in other contexts.

**Supplementary Information:**

The online version contains supplementary material available at 10.1186/s12913-026-14555-6.

## Background

Primary health care (PHC) is the foundation for universal and high-quality health care coverage. It is estimated that 90% of essential services can be offered at the PHC level, thereby reducing costs to the health system and to patients and improving access to care and service coverage [1, 2]. In emergency settings in particular – conflict and natural disasters – where health risks are exacerbated, PHC is vital to supporting emergency preparedness and resilience of health care provision [3]. As the point of care closest to patients, PHC providers are best placed to understand and monitor the overall needs of their local population, to identify vulnerable groups, to ensure continuous access to essential services, and to preserve community engagement and trust in these services. PHC is also best placed to recognise new health risks or needs that may emerge during an emergency. In conflict-affected settings, the maintenance of government-led delivery of essential health services has been proposed as necessary for sustaining access to care and social cohesion, thus improving health outcomes and supporting government stability [4]. However, maintaining essential PHC services in times of crisis is challenging. Local manufacturing is reduced, supply chains are disrupted, and the national currency is devalued, resulting in increased costs of service delivery. Population movements lead to changes in service demand and health worker availability across different geographic areas. Demands on PHC also surge in the immediate aftermath of a crisis, and often peak again in the months that follow, imposing a prolonged strain on already weakened systems [3, 5, 6]. Sustaining financial incentives for quality PHC services and ensuring equity of financing across all PHC facilities – both considered crucial for effective PHC service provision [7, 8] – becomes particularly challenging as government budgets are reduced and dependence on donor funding increases [9, 10].

Despite an acknowledgement of the importance of PHC to health and resilience in emergency settings, there is very little documented evidence of the impact of crises on PHC care service disruptions, demand, and financing. The WHO Health Resources Availability Mapping System (HeRAMS) [11] is a standardized tool for monitoring the availability of health services during an emergency response and includes data collection on overall functionality of PHC services. However, it does not include granular assessments of the impact on PHC service provision. As a result, there is little evidence to inform strengthening and rebuilding PHC in ways that ensure equitable access to high quality and appropriate PHC services [4, 9, 10, 12, 13]. We aimed to contribute to addressing this gap in evidence by analysing the impact of war on PHC in Ukraine.

### PHC in Ukraine

In Ukraine, Russia’s full-scale invasion coincided with the early stages of a series of health system reforms, initiated in 2016 [14, 15]. Central to the reforms was the formation of a national strategic purchasing agency (the National Health Service of Ukraine, or NHSU). Public (57%) and private (43%) [1] PHC providers were invited to enter into contract with the NHSU and be paid on a capitation basis, with explicit and unified contract terms [16]. Ukrainians were encouraged to select and register (or “sign a declaration”) with a PHC provider in order to access an explicit benefit package (the Programme of Medical Guarantees (PMG)) which outlined essential primary care services that contracted PHCs were obliged to offer. In brief, these include consultations, interventions, laboratory and diagnostic examinations for common and chronic conditions (general blood test with leukocyte formula, general analysis of urine, blood glucose testing, total cholesterol testing, rapid tests for pregnancy, troponin, HIV, viral hepatitis B and C, test for the severe acute respiratory syndrome coronavirus disease (SARS-CoV-2) antigen), preventive screening, vaccination, antenatal care of noncomplicated pregnancy, healthy child development monitoring and certain types of emergency and palliative care. PHC was envisioned to be the first point of care for patients. By 2023, 80% percent of the population had registered to a specific provider [16]. These reforms were supported by the introduction of a national e-Health system for management of patient registration, prescriptions, and referrals and an Affordable Medicines Programme to cover selected essential medicines [17, 18]. State financing of PHC was increased from 3 billion UAH (approx. $107 million USD) in 2018 to 23 billion UAH (approx. $626 million USD) in 2023 from centrally pooled funding [19]. Increased resources in Ukraine’s PHC system enabled it to have a significant role in responding to the Covid-19 pandemic [20], including through PHC coordination of testing and nation-wide vaccine administration.

However, significant challenges remain. While the doctor-to-patient ratio in Ukraine (approximately 40 per 10,000) is quite similar to EU countries, Ukraine has a significantly lower density of nurses (42 per 10000 compared to 91 per 10000), midwives, dentists, and physiotherapists. While PHC providers can include nurses working alongside general practitioners and pediatricians, the role of nurses has historically been limited (although reforms are underway to expand their role to more clinical tasks). There is also geographic inequality - around 30% of the population of Ukraine lives in rural areas but only 17% of PHC doctors and 7% of all nurses work in rural areas. More than half of PHC doctors are above 50 years of age and 29% are above 60. The country also continues to battle with a high burden of chronic illness. For example, Ukraine already had one of the highest mortality rates from ischemic heart disease in the world (399/100,000) before the full-scale invasion in 2022 (compared to 96/100,000 in the UK, for e.g.). The condition has worsened since - NHSU reports that the prevalence of ischemic heart disease has increased by 31% since 2021. The prevalence of common mental disorders is also high – in 2023, 36.3% of Ukrainians met the requirements for any mental disorder (including anxiety, depression, or alcohol use disorder). Starting from 2022, the full-scale invasion of Ukraine by the Russian Federation has caused extensive damage and disruption to the health system in Ukraine, including to PHC [21]. As of the end of May 2024, the World Health Organization (WHO) had verified 1820 attacks on health care including attacks affecting PHC facilities [22]. Many of these facilities are in areas with active hostilities, where access to health care and medications is already heavily disrupted and tracking population movements and access to care is very challenging [23, 24]. Over 3.4 million people in Ukraine have been internally displaced and 6.5 million have left the country as refugees [25, 26].

## Methods

Our aim was to explore the extent to which PHC service provision in Ukraine were impacted by the full-scale invasion in terms of patient profiles and health needs, services demanded, health worker burden, and disruptions to facility operations and financing, and whether this impact varied by regions. We used a mixed-methods approach, drawing on data from a survey and qualitative interviews.

### Survey

The survey sample was a sub-sample of an earlier survey that investigated costs incurred by PHC providers. That study sample was selected from the 2226 providers in the country that had contracts with the NHSU for PHC services in 2021. Selection criteria were developed to ensure representation of different types of providers (publicly or privately-owned) from different regions (shown in Table 1). Further details on the sample size calculation for the costing study and other sampling details have been previously published [27]. For the present survey, in order to ensure a maximum theoretical sample error of 10%, with a 95% confidence level, we targeted 100 providers. Quota sampling was used to ensure a maximum representative sample of existing PHC facilities, their locations, ownership, sizes and types, but also considering provider willingness to contribute. A total of 86 providers accepted the invitation to participate. Survey data were collected using an online questionnaire developed specifically for this study and completed in January-February 2023.

**Table 1:** Selection criteria for PHC provider participants in survey

Category	Eligibility criterion
Ownership	Publicly-owned providers and fizychna osoba-pidpryemets (FOP) (private legal entity)^*^
Type of institution	PHC providers contracted only for the PHC benefit package by the National Health Service of Ukraine**
Number of patients per doctor	900–2000***
Structure by regions (based on actual proportion of PHC providers)	Central − 20% Eastern − 18% Northern − 16% Southern − 13% Western − 33%
Structure of rural/urban area providers	Public providers: 22% rural; 78% urban FOP 12% rural; 88% urban

* Private providers contracted by the NHSU for the PHC benefit package include FOPs which are individual entrepreneurs or small or medium-sized enterprises and larger private entities. For the survey, only FOPs were included as the larger private entities may also provide other services beyond the NHSU funded Program of Medical Guarantees

** Until 2023 the NHSU could contract any health care provider for the PHC package, including outpatient specialist care providers and hospitals. As the study focuses on PHC services, the sample includes only providers that are not contracted for any services by NHSU other than PHC services

*** The range of 900–2000 patients was used to exclude providers providing only paediatric care where the max threshold per doctor is 900 and those providers exceeding the 2000 patient upper limit set by the NHSU for PHC providers (any number of patients exceeding this limit are reimbursed at a lower rate to disincentivise high patient to doctor rate)

**Fig. 1:**
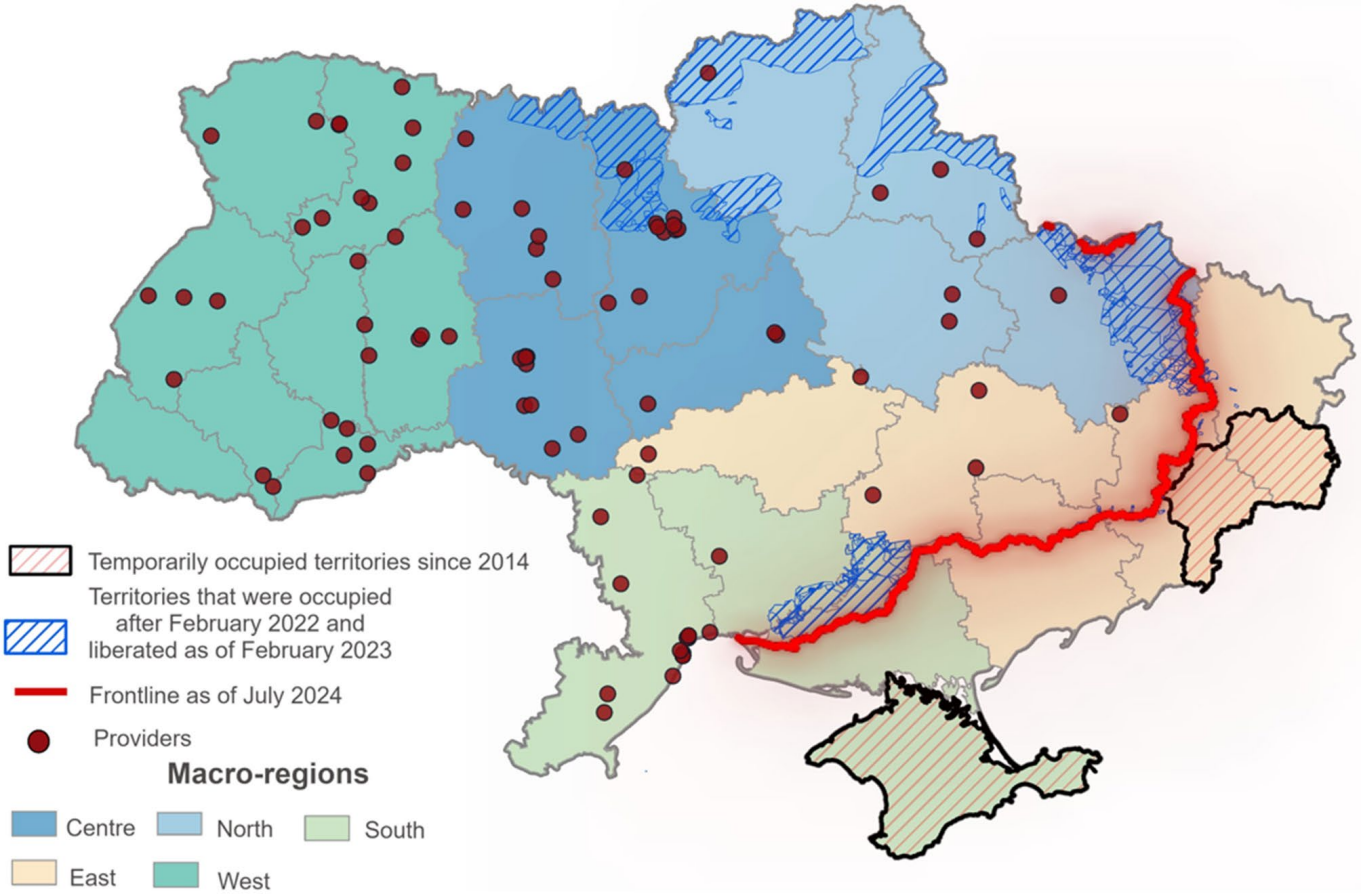
Map of Primary Health Care providers participating in online survey (*n* = 86)

Each provider determined on their own who within the facility would complete each component of the survey, depending on who was most knowledgeable about each topic. Typically, this was the general manager, chief accountant, medical director, or chief nurse. The locations of the providers are shown in a map as Fig. 1. The questionnaire covered the overall impact of the war on PHC facilities, changes in patient numbers, profiles, health and service needs, health worker time, and the nature of specific disruptions to service provision and financing. The selected survey questions used for this study are shown in Appendix 1. We analysed survey results descriptively to estimate the prevalence of each variable and calculated 95% confidence intervals (CIs) for each estimate using the Wilson score interval method.

### Qualitative interviews

Forty-one PHCs were from the most conflict affected areas of Dnipro, Donetsk, Zhytomyr, Kyiv, Mykolaiv, Odesa, Sumy, Kharkiv or Chernihiv. The survey identified 32 providers that reported an impact of the full-scale invasion on their services from which 21 which were from the most conflict affected areas. From these 21 providers, 15 were randomly selected and invited to participate in in-depth semi-structured qualitative interviews in March 2023. The sample size was determined by the principle of data saturation, where further interviews did not lead to new themes emerging. The topic guides were developed with input from national and international experts on PHC service delivery. They included questions about changes in patient profiles, services and service delivery, and revenue and expenditures. Interviews were conducted in Ukrainian, over the phone by a single trained interviewer, and lasted approximately 45 min each. They were audio recorded and transcribed. Interviews were analysed thematically and compared to the survey results to identify and further explain areas of convergence or divergence. An English-language translation of the interview topic guide is included as Appendix 2. Informed consent was obtained from all interview participants and the informed consent form is included as Appendix 3.

## Results

The descriptive characteristics of included PHCs is outlined in Table 2.

**Table 2:** PHCs included in survey

Macroregions	Communal	Private (FOP)	Total
Western	23	6	29
Southern	5	11	16
Northern	6	1	7
Eastern	4	2	6
Central	22	6	28
Total	60	26	86

### Overall impact and causes of disruption

**Fig. 2:**
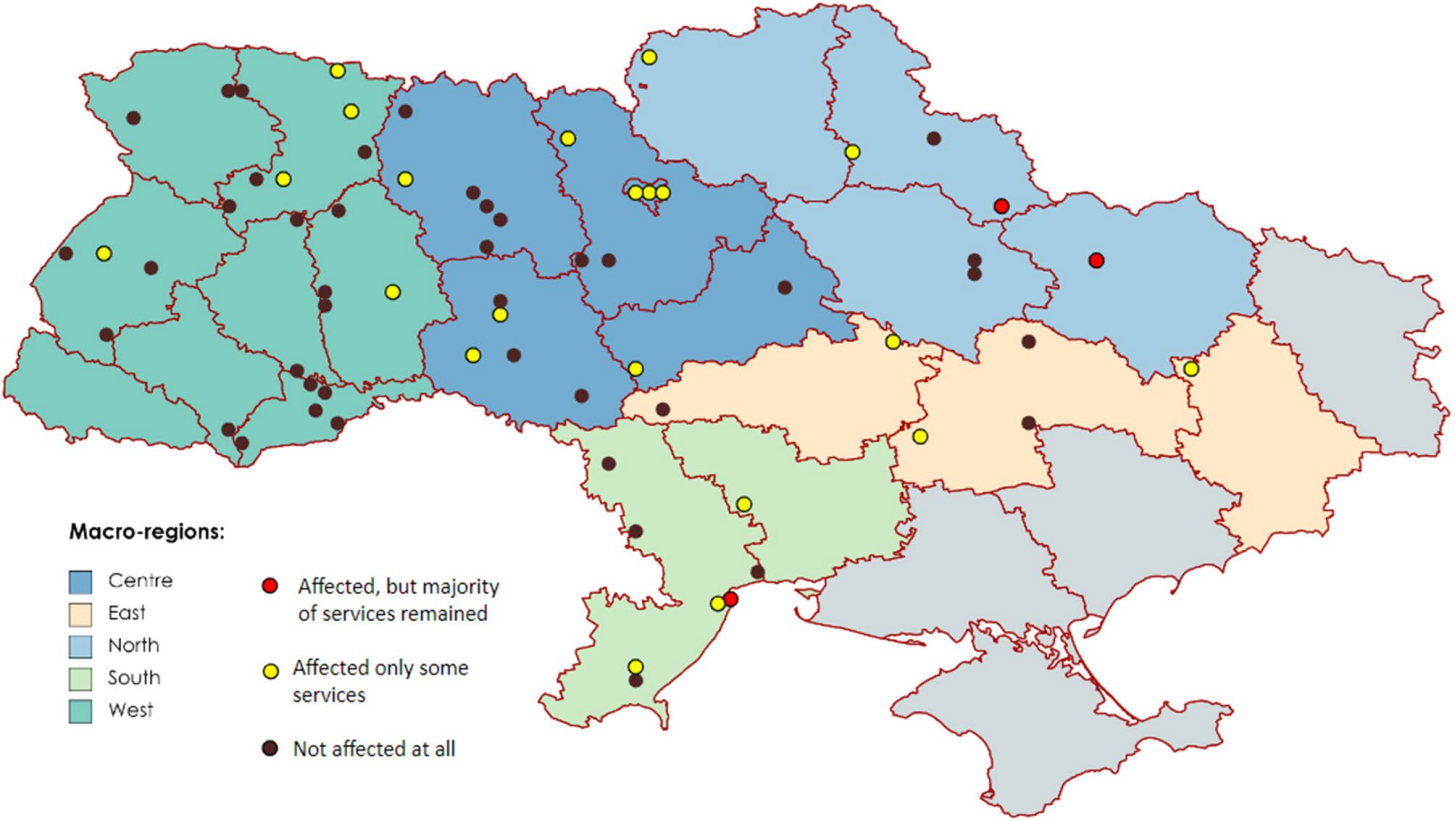
Map with locations of PHC facilities at oblast level affected by the full-scale invasion

In the survey, a total of 37% (95%CI: 28%-48%)) of PHC providers reported that their facility was affected by the full-scale invasion. Figure 2 shows facilities reporting an impact by geographic location.

However, all these facilities reported being able to continue providing the majority of services. No PHC facilities included in the survey reported being so heavily affected that it was impossible to provide any services. Data from qualitative interviews provided a nuanced picture of the impact of the war on PHC facilities’ capacity to continue service delivery. These data highlighted a much greater impact in the immediate aftermath of the full-scale invasion, with service provision stabilising after 2–3 months as facilities adjusted to new circumstances. For example, from providers in Kharkiv and Rivne:

It is important to note the different periods here because a year has passed and different periods were affected differently by the full-scale invasion. In the first months, when it was March-April, we had, how to say it correctly, idleness. We went to work, but there were almost no patients, because most of the patients had left. Only some services were available. More activity began in mid-April. Around May, we started to work in the same mode as before.

It was difficult to adapt, especially for doctors. The reception schedule has changed because there was a small influx of internally displaced persons at first. Air raids - since there is no shelter in our facility, the closest to us is roughly a kilometre and a half, and you understand that every time there is an alert, you take everyone out, one and a half kilometres in one direction, one and a half kilometres in the other direction. As a result, the entire appointment goes astray, people are in queues, doctors switched to telephone communication. Over time, somewhere within a few months, it somehow more or less stabilized.

In response to the survey question of the impact of various disruptions to provision of PHC services, the most reported disruptions were electricity cuts (76% (66%-83%)) and depreciation of the national currency/increases in prices (72% (62%-80%)). An increase in number of patients, medical workforce shortages and heat supply cuts were also common causes of disruptions, reported by 33% (24%-43%), 31% (23%-42%) and 30% (22%-41%) providers, respectively. Interestingly, three providers who declared that the war had no impact on their activities, reported some impact when asked about specific disruptions and the impact those disruptions had on service provision.

### Change in number of patients or number of visits

**Fig. 3:**
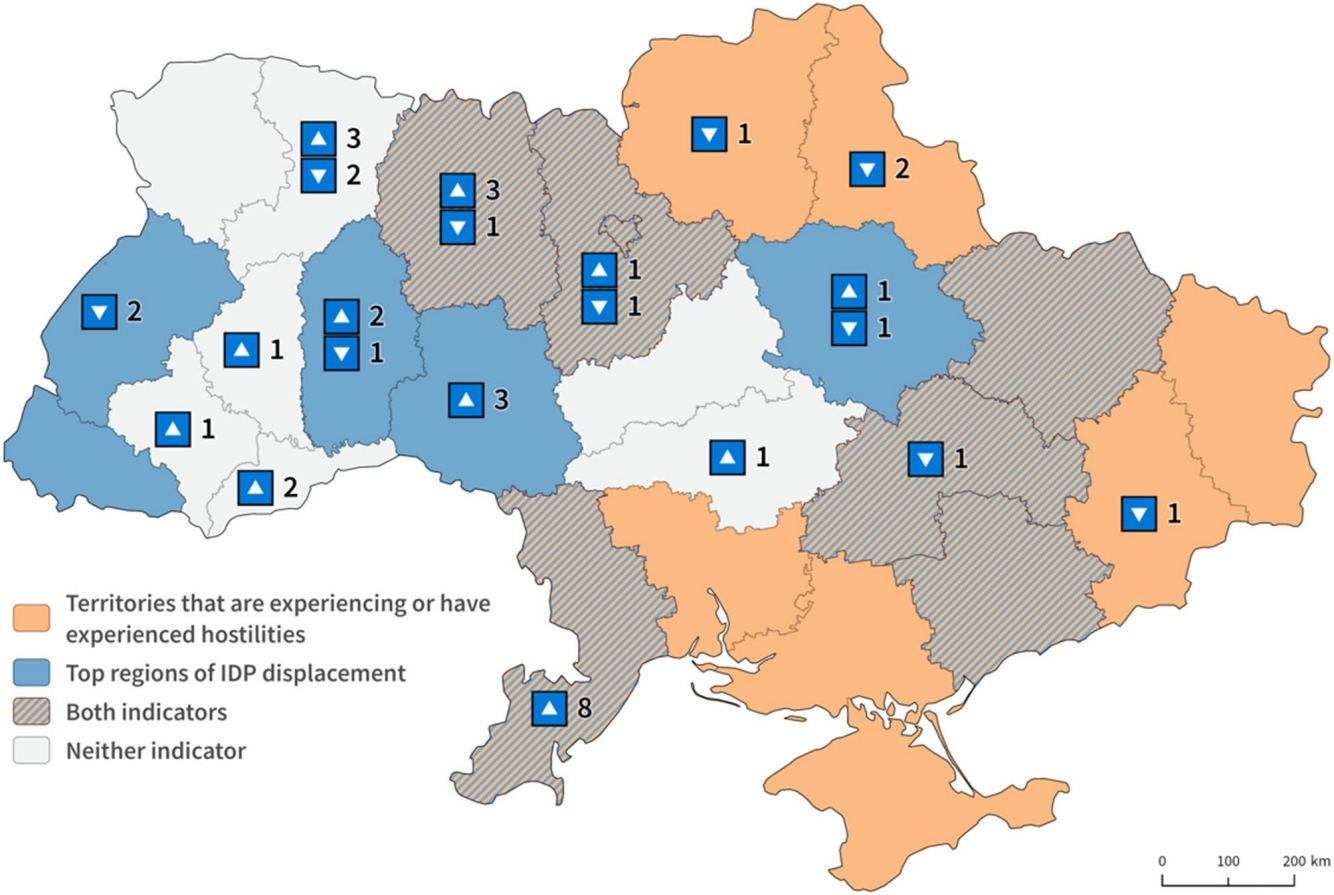
PHC providers reporting changes in number of patients with declarations since February 24, 2022. IDPs = internally displaced people; “Top regions for IDP displacement” = those regions to which the most IDPs have moved. (Arrows pointing up indicate the number of PHCs in that region reporting an increase in number of patients with declarations) arrows pointing down indicate the number of PHCs in that region reporting a decrease

Thirty-percent (22%-41%) of providers indicated that the number of patients who signed declarations at their facility increased since the full-scale invasion on February 24, 2022; 15% (9%-24%) indicated that the number of patients registered decreased; one provider was unable to respond to this question, and the remaining providers reported no change in patient numbers. Figure 3 shows the number of facilities reporting increases or decreases in patients according to location.

In response to the question of whether the number of visits by patients with declarations changed since February 24, 2022, 52% (42%-63%)) indicated that the number of visits had changed significantly. Among them, 28% (17%-34%) noted that the number of visits has increased, and 24% (20%-38%) that the number of visits has decreased. In the qualitative interviews, the concerns of PHCs providers about the potential impact of decreased visits was evidence. For example:

Due to the decrease in the number of visits, we did not examine enough patients for the package of tuberculosis treatment at primary care. At the beginning of 2023, we see an increase in the incidence of tuberculosis. And an increase in the incidence of tuberculosis among contact children. This worries us a lot.

### Change in reason for seeking care and type of consultation provided

**Fig. 4:**
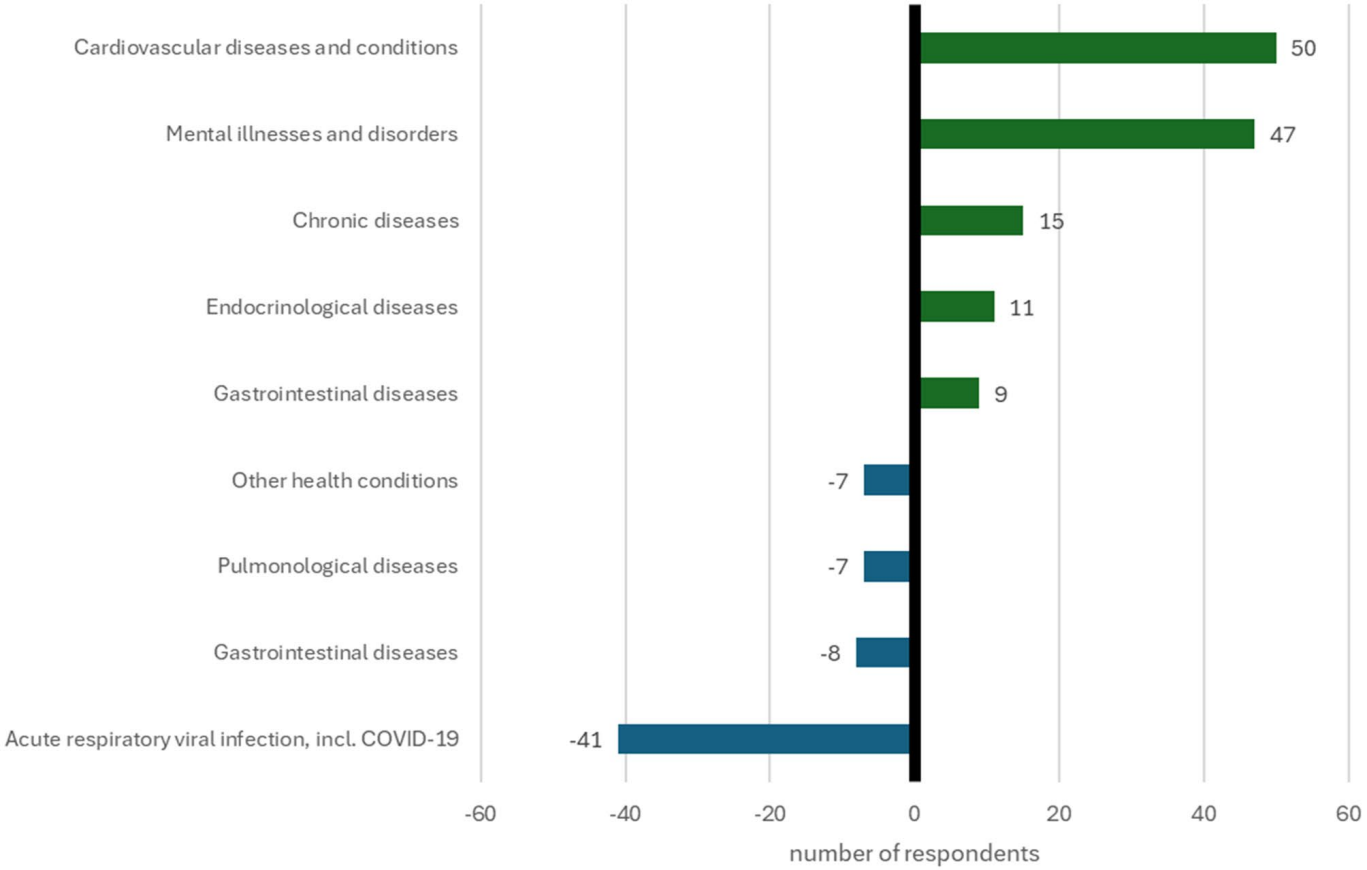
Conditions that either increased or decreased as reason for patient visits to PHC providers. The “chronic diseases” category includes chronic illnesses not captured by the other categories, including back pain, chronic bronchitis, and exacerbations of existing chronic conditions

Almost all providers indicated a change in the reason patients were seeking care. Figure 4 shows the conditions that were reported as increasingly common reasons for PHC visits, and those that decreased. The most commonly reported increased medical need from current patients were cardiovascular diseases, reported by 58% (48%-68%) of PHC facilities. The free text responses included “hypertensive crisis”, “cardiac pathology”, “hypertensive conditions”, “hypertensive disease”, “ischemic heart disease” and “increased blood pressure”. This was followed by an increase in mental illnesses and disorders, reported by 55% (44%-65%) of PHC providers. These included anxiety disorders, depression, panic attacks, insomnia, nervous disorders, stress conditions, phobias, and PTSD. The most reported category that decreased as the reason for visits was acute respiratory viral conditions, including COVID-19.

Our qualitative data highlighted important details about these changes. Several providers emphasised not only the increase in care-seeking for cardiovascular disease and related conditions, but also that this care was increasingly sought by younger people. As described by one provider in Odesa:

The number of acute disorders of cerebral blood circulation has increased. Strokes have become younger. We have several cases of myocardial infarction just over the age of 35. During my 20-year work before the war, I had only 1 case of acute myocardial infarction in a 36-year-old man. But now there are several cases….In addition, hypertensive disease cases have become younger and more vicious. They have more pronounced crises.

**Fig. 5:**
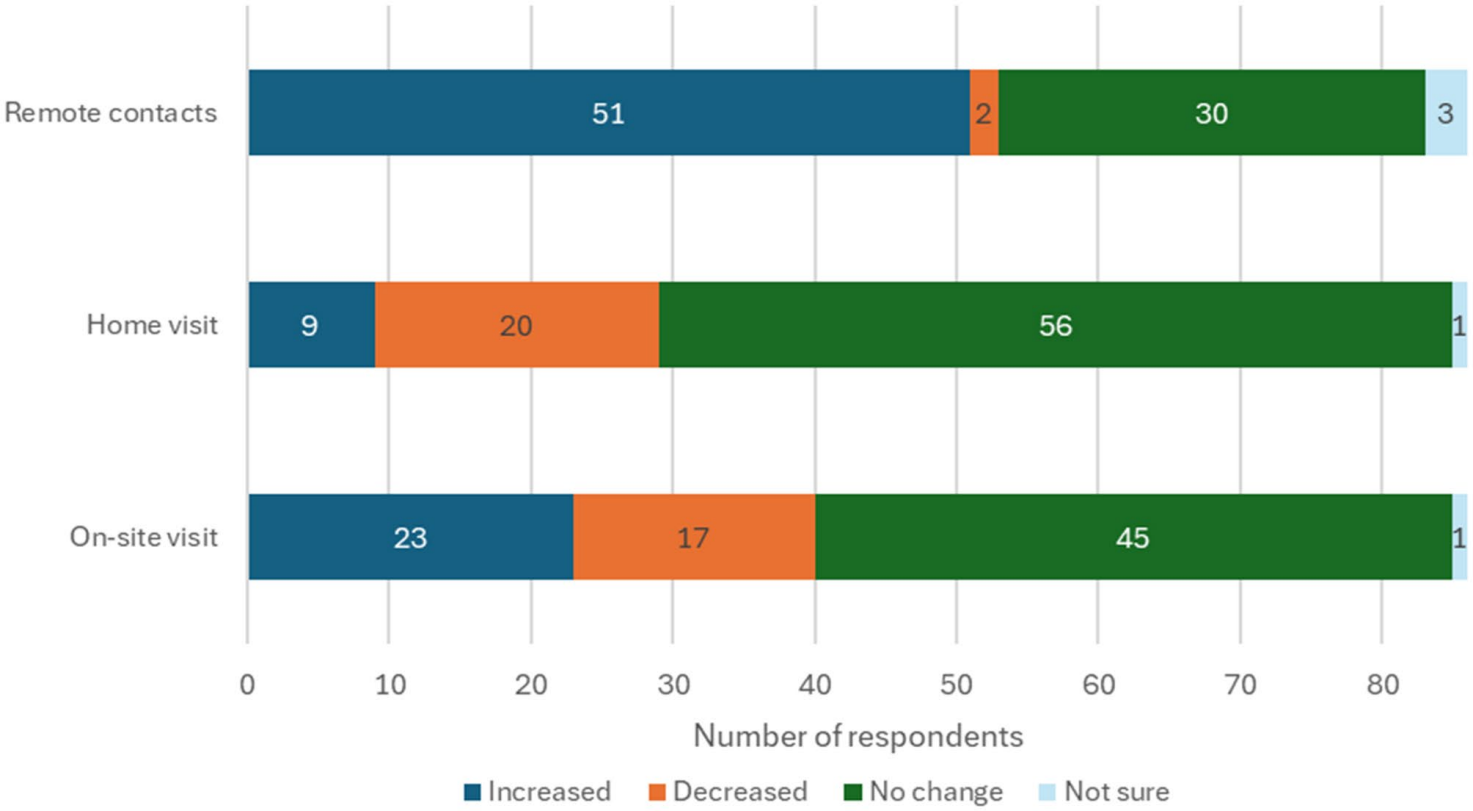
Changes in types of consultations among patients with declarations

In response to questions about changes in type of interaction or consultation with patients, 59% (49%-69%) of PHC providers reported an increase in remote consultations, and only 2% (0.6%-8%) reported a decrease in this type of consultation (Fig. 5/Table 3). At home and PHC facility-based consultations were reported to both increase and decrease by different facilities.

**Table 3:** Changes in types of consultations among patients with declarations

	Remote contacts	Home visit	On-site visit
Increased	51	9	23
Decreased	2	20	17
No change	30	56	45
Not sure	3	1	1

### Change in provider revenues and resource needs

Providers were also asked about changes in their revenue sources. Most providers (71% (61%-79%)) indicated that the revenues they received from NHSU did not change. Among those facilities that did report a change in their revenues, the nature of the change depended on the type of ownership − 23% (11%-42%) of private providers observed a decline in paid services, but only 4% (*n* = 2 providers) of public providers did so. In either case, providers continued to provide services regardless of whether they would be paid. As described by one provider:

There were people who were temporarily displaced, had status or did not have status and we made a decision (by order) that we will serve people who do not have declarations with us, without any additional costs on their part. Because in wartime conditions, people simply have to help each other in a humane way, particularly since we had sufficient supplies for emergency medical care. I mean medicines, rapid tests, laboratory tests.

Only 8% (2%-24%) of private providers indicated an increase in revenues from humanitarian or charitable aid, while 97% (87%-99%) of public providers indicated an increase in these sources. No private providers reported increased funding from local authorities (e.g. municipal or village councils), while 20% (12%-32%) of public providers reported an increase in funding from local authorities.

**Fig. 6:**
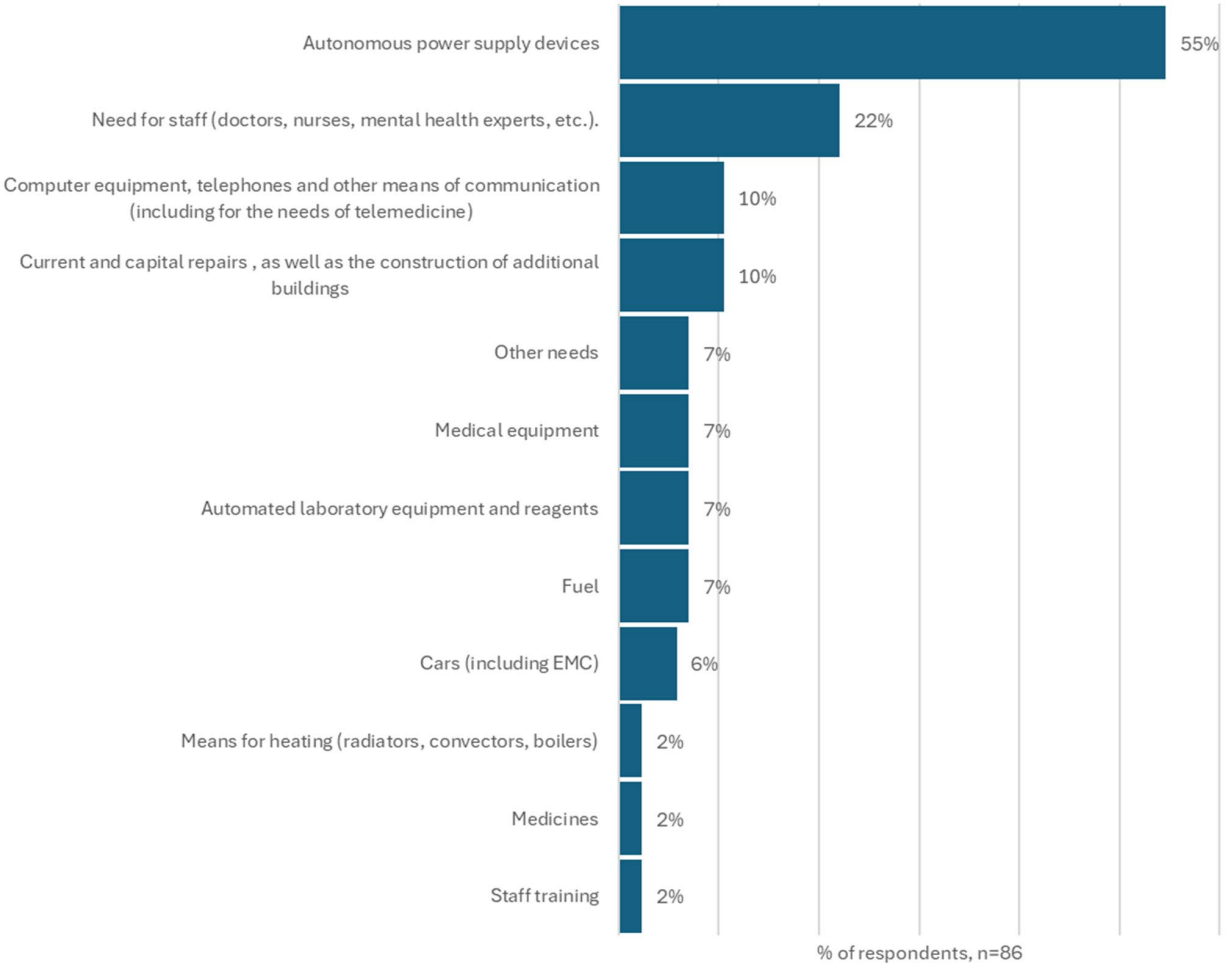
Provider self-reported needs as a result of war. “Other needs” includes financial, supply, and regulatory support needs that do not directly fall under the separate categories of equipment, personnel, or infrastructure. These include reimbursement for power sources independently purchased, masks, disinfectants, and similar supplies, and legal regulation of certain aspects of facility operations and patient access to care and medications

Finally, providers were asked about the impact of the war on resource needs. As a direct impact of electricity cuts, the majority of providers indicated a need for an autonomous power supply device (55% (44%-65%). 22% (15%-32%) of providers reported a need for staff including doctors and nurses, but particularly mental health experts. The need for capital repairs and construction, computers, and phones were also commonly reported (Fig. 6).

### Change in PHC health worker hours

**Fig. 7:**
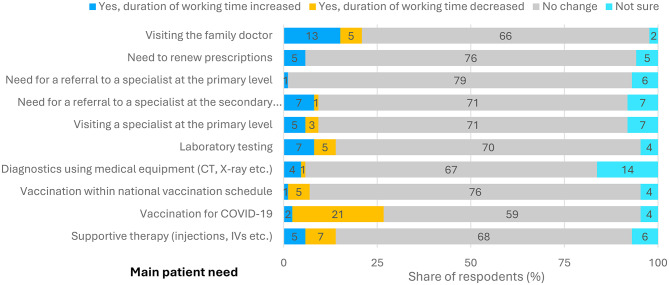
Changes in working hours of medical personnel

When asked if the duration of working time increased or decreased on specific tasks, the patient need for which most PHC workers experienced increased time was consultations (or “visiting the family doctor”) – 13% (7%-21%) reporting increased time. The patient need for which most reported decreased working time was vaccinations – 21% (14%-31%) reporting decreased time (See Fig. 7).

The qualitative interviews highlighted the impact of electricity cuts in particular on working hours. As one provider in Cherkasy described:

Turning off the electricity also affected the fact that doctors had to stay longer at work in order to partially conduct medical records of the patients they had provided services to in the morning. We have a rural area. And very often the electricity was cut from 8 to 12 in the morning, which is precisely the time when the doctor is actively conducting appointments. However, the referral cannot be issued, they cannot do anything.

## Discussion

Using a mix of online survey and qualitative interviews, we were able to assess the impact of the Russian Federation’s full-scale invasion on PHC service provision in Ukraine. These findings have important implications for strengthening preparedness and resilience of PHC in Ukraine, particularly in relation to what services are included in PHC and how they are delivered and financed.

### The impact of war on service provision

Our study found that no PHC providers were so affected that they were unable to provide services. This finding is in line with that of the WHO HeRAMS conducted from November 2022 to May 2023 which found that 90% of PHC facilities did not sustain any damage, 93% of PHC providers’ equipment remained intact, and 93% of PHCs facilities were fully functional [28]. It is also in line with the WHO Health Needs Assessment (HNA) survey conducted among a cross-sectional sample of Ukrainian adults in April 2023, in which 96% of participants reported that their PHC provider was functioning (although there were differences among areas with active hostilities (94%), areas that had experienced hostilities in the past (96%) and the rest of the country (97%) [29]. A follow up HeRAMS conducted in 2024 found that 97% of PHCs were fully functional [30]. This is consistent with data from our qualitative interviews which suggested that services stabilised over time.

The evidence stability of service provision supports earlier assertions that Ukraine’s health system has demonstrated resilience in war time. Earlier work has underscored the importance of government efforts to maintain public financing of PHC, strategic adaptations to service organisation during the COVID-19 pandemic including the large-scale use of digital communication and skills training, and PHC-level managerial and financial autonomy to respond quickly and efficiently to crisis [10]. Despite this overall success, specific impacts should still be carefully considered to ensure continued resilience.

### Impact on health needs

Our study also revealed that providers identified an increase in demand for care for cardiovascular disease, particularly among younger adults. This increase may even be an underestimate of need, given findings from the HNA that suggest people with chronic conditions in the most conflict-affected regions forego seeking care for their conditions [29]. The findings are consistent with international evidence of the increase in incidence of these diseases and associated complications in crisis-affected settings, often due to disruption of health services and medicine supply and changes in diet and lifestyle (e.g. increased smoking), all of which can introduce or exacerbate existing risk factor conditions such as hypertension and dyslipidaemia [31]. Where health care systems are overwhelmed with acute emergency health needs, routine prevention and management of chronic diseases is often overlooked, leading to more costly and serious complications.

This finding highlights the importance of including essential services for primary prevention of cardiovascular disease within the Programme of Medical Guarantees at the PHC-level funded by the NHSU. These services should, at minimum, include those outlined in the national guidelines, being developed by the Ministry of Health with support from the WHO [32, 33]. For example, the benefit package currently excludes some services like serum creatinine, urine microalbumin or lipid profile tests, which are identified as essential in the WHO Package of Essential Non-Communicable Disease Interventions [34]. Furthermore, the utilization of basic diagnostic test is low at the PHC level [35] which may be a barrier to effective chronic care management.

We also found an increased need for care for mental illnesses and disorders, which is also consistent with evidence from other crises settings and research from Ukraine [29]. In response to the increased need for mental health care in Ukraine, a new mental health service package was gradually introduced in 2022 into the Programme of Medical Guarantees at the PHC level [36], to be universally implemented by 2025. In parallel, the WHO is supporting the Ministry of Health to strengthen the capacity of all PHC health workers in priority mental health care interventions outlined in the WHO Mental Health Gap Action Programme (mhGAP) Intervention Guide [37]. As of June 2024, 13.5% of PHC doctors and 8.4% of PHC nurses in Ukraine had been trained in mhGAP. The MoH also plans to implement “psychological relief rooms” in PHC facilities where family physicians will provide basic psychological assistance free of charge.

Any training of health professionals must be complemented with ongoing support that recognises the impact that the war and changing health care demands has had on their workload and well-being. In Ukraine, the WHO, MoH and other partners have implemented several initiatives to address staff shortages and burnout among medical personnel. This has included, for example, financial incentives to retain skilled professionals and psychosocial support programmes, particularly in high-risk areas where health workers face immense pressure and longer work hours. The impact of these initiatives on health worker retention and well-being should be evaluated to inform future efforts in Ukraine and other conflict-affected settings.

### Adaptation to PHC service delivery

Our study showed that roughly half of providers experienced changes in number of visits and the same number reported an increase in remote consultations. These results are consistent with findings from the HNA showing significant increases in reported remote contacts with family doctors among the public, including through instant messaging and online calls [29]. The increased dependence on remote consultations mirrors the experience of other European countries [38], and other crisis affected settings [39], during the COVID-19 pandemic. For example, by December 2020, 10 months into the pandemic, telephone consultations were available in at least 39 countries in Europe and consultations via e-mail, video, online chat, and Electronic Health Records portals were commonly used [38]. The persistence of the use of remote consultations in many settings may be due to evidence suggesting their potential effectiveness as one component of disease management for people with multiple but stable chronic conditions including cardiovascular disease, diabetes, hypertension, and cancer with a high level of patient satisfaction and reduced cost [40–44]. Remote service delivery may be especially promising as a means of improving access to care in the most conflict-affected regions of Ukraine. However, challenges with remote consultations have also been identified and are relevant to the Ukrainian context. They include variation in access to the required technology and internet, privacy concerns and data breaches, and uncertainties about medical liability and standards of care. Some work has also questioned the appropriateness of remote consultations for older populations, those who lack digital literacy, and the poor, although other work has shown that these need not be barriers to use of remote care [45]. There is also the risk of health issues being missed when patients do not attend in person, and specific in person services being under-provided. These issues are important to consider if remote consultations are to be adopted as a long-term approach, to ensure equitable and high-quality care. If remote consultations are to be used, they could be supported by regular outreach services to allow for in person physical examinations and review of prescription availability and adherence [46].

### Financing PHC service provision

Currently, the PHC system in Ukraine is mostly publicly funded by the NHSU, financed on a per capita basis with some providers receiving additional revenues from local governments and donors. Many Ukrainians residing outside the country or internally displaced maintain their declarations with their PHC provider and providers continue receiving payments from NHSU for these patients, even where these patients may have coverage in their new place of residence. As a result, revenues for these facilities did not change significantly. Some people may continue to access services remotely, but the service quality and the underlying costs to providers to deliver services remotely is different from in person consultations. This raises important questions about how to fairly and efficiently allocate the scarce resources available for PHC, and specifically whether a system where providers revenues are relying mostly on public funding defined by number of declared populations is the most suitable in a context of crisis and population displacement. Many years into the ongoing hostile invasion, the country may not be able to afford paying on behalf of patients who have left the country. On the other hand, it is difficult for NHSU to assess what is the valid underlying population number for patient declarations forming the PHC budget. Thus, in 2025 the country executed a declaration validation process, making PHC providers to confirm persons presence in the country. But, any changes in patient declarations will impact provider revenues, which incentivizes providers to focus on optimizing their patient count rather than focusing on the number and quality of services provided. In addition, there is a high risk of reduced revenues in most conflict-affected areas, as these areas have suffered the largest emigration. As a result of the population validation, PHC providers especially in conflict affected settings may lose significant share of their revenues and may be financially unsustainable. To mitigate this risk, evidence suggests that equitable financing of PHC should ensure stable provider revenues and be paid based on a context-specific blended payment model with capitation at its centre [47] In Ukraine, lump-sum payments could be considered to offset loss in revenues due to a limited number of people in rural and conflict-affected areas, ensuring sustained access to services for people in these areas. The payment model could be blended with a fee-for-service component to limit incentives for under-utilization and a system of top-up payments could be considered to incentivize providers to improve NCD management. To support more comprehensive PHC services in general, including responding to the increased need for NCD and mental health management will require increased funding for PHC. This must include increased funding for outpatient medicines, given recent evidence of gaps in access to medicines in Ukraine and reductions in households’ capacity to pay since the start of the full-scale invasion [48]. Important positive steps have been made, such as expanding the list of medicines covered by Affordable Medicines Programme, including a focus on NCDs. There is a need to continue on this path in contexts like Ukraine, where resources are increasingly scarce, prioritizing funding for known cost-effective interventions such as core PHC services including diagnostics and outpatient medicines. Funding must also be allocated equitably, given the vast evidence that an equitably financed PHC system delivers the best value for money [47]. Our findings suggest that privately-owned PHC providers are more likely to suffer decreases in NHSU funding and less likely to receive increased funding from alternative sources during a crisis, suggesting a need for reforms to provider payment structures. Any approach to increased funding must be careful to include costs for emergency-related needs such as those identified in our study, to ensure PHC facilities can deliver their basic functions e.g. back-up generators to maintain refrigeration of vaccines [27].

### Limitations

Our findings should be interpreted in light of some limitations. First, the survey responses rely on self-report which may be vulnerable to recall bias, potentially leading to forgotten impacts of the war and an overall underestimate. To try to address recall bias, questions were included about specific areas of impact, in order to prompt recall where overall impact might have been underestimated. Self-reported impacts on service provision may also be limited by concerns among providers that a reported reduction in service provision may result in reduction in funding allocated to them from NHSU. The result of this would be an overall underestimation of impacts on service provision. Second, the data on which this study is based include limited representation from PHCs in the most affected or occupied areas of Ukraine, due to unreliable internet coverage and electricity supply, and restricted access to these locations. As data from the HeRAMS analysis [24] suggests that facilities in heavily conflict affected or occupied areas may be the most impacted in terms of service provision, future studies may consider exploring means of collecting detailed data from these hardest to reach facilities (for e.g. shortened version of surveys conducted over mobile phone), drawing on lessons from adaptations made in other humanitarian crises [49]. Third, our data are cross-sectional and thus don’t allow us to look at changes over time. While our qualitative data addressed this limitation to some degree, providing insight on dynamics in the impact of the war on PHC providers from the onset of the full-scale invasion to the time of data collection, it will still be important to consider repeating the survey to identify longer term trends in the impact of the conflict on primary care.

## Conclusion

There is limited documented evidence of the impact of conflict on PHC providers. This study highlights several impacts of the war on PHC in Ukraine. These are likely an underestimate of the true impact due to the omission of facilities in the most affected or occupied areas of the country. Nevertheless, they raise key considerations for service delivery and financing that are important for strengthening PHC resilience in this and future crises. It is crucial that the PHC benefit package aligns with clinical guidelines, particularly for cardiovascular diseases and mental health, and that the PHC workforce be trained in provision of essential services for these conditions. As demands increase, health workers must be given appropriate support, including for psychological well-being. Increases in remote consultations must be closely monitored for quality and potentially combined with physical outreach services to ensure equity in access. Payment systems must adjust to new circumstances – while alternative financing sources may cover new costs that arise as a result of the war, there is a need to ensure equity of financing regardless of provider ownership. With these considerations in mind, Ukraine can continue to prioritise PHC as the most inclusive, effective, and efficient approach to achieving universal health coverage in times of war and peace.

## Supplementary Information

Below is the link to the electronic supplementary material.

Supplementary Material 1

## Data Availability

The dataset used during this study is available from the authors upon reasonable request and permission from the World Health Organization Country Office in Ukraine.
