# Supplementary material for: The impact of war on Primary Health Care in Ukraine: a cross-sectional survey and qualitative interviews with service providers
Source: BMC Health Serv Res. 2026 May 9;26:901. doi: 10.1186/s12913-026-14555-6 (PMC13326145; doi:10.1186/s12913-026-14555-6)
Supplement: Supplementary file 1 — Supplementary Material 1 [file 12913_2026_14555_MOESM1_ESM.docx]

Supplementary material

Appendix 1: Survey questions

1.1 In your opinion, on a range from 1 to 5, how much your facility was affected since 24 February 2022 till the current time, where:

5 = being heavily affected in a way that it was not possible to serve patients

4 = heavily affected but some services remained

3 = affected, but majority of services remained

2 = affected only some services

1 = did not affect at all

| 1 | 2 | 3 | 4 | 5 | NOT SURE |
| --- | --- | --- | --- | --- | --- |
|  |  |  |  |  |  |

Responders should tick the right block.

**Patient profile**

2.1 Did the number of patients with declarations change significantly since 24 February 2022?

1. Yes, increased (please, specify the approximate percentage change)
2. Yes, decreased (please, specify the approximate percentage change)
3. No
4. Not sure


   2.2 Did the number of patients' visits (with or without declarations) change significantly since 24 February 2022?

| Types of patients | Yes, increased | Yes, decreased | No | Not sure |
| --- | --- | --- | --- | --- |
| Patients with declarations |  |  |  |  |
| Patients without declarations |  |  |  |  |

Responders should tick the right block for each type of the patients.

If the responder answers 'Yes, increased' or 'Yes, decreased' on patients without declarations, please ask the following question.

2.2.1 Please specify the approximate percentage change.

2.2.2 Did the number of internally displaced people (IDPs) among patients without declarations change significantly since 24 February 2022?

1. Yes, increased (please, specify the approximate percentage change)
2. Yes, decreased (please, specify the approximate percentage change)
3. No
4. Not sure

   2.3 Did the age and sex distribution within patients' visits change since 24 February 2022?
5. Yes
6. No
7. Not sure

   If the responder answers Yes to the Question 2.3, please ask the questions 2.3.1 and 2.3.2.

   2.3.1 Please, specify the number of patients visiting your facility during the period from 24 February 2022 to 1 December 2022 by the following age and sex groups:

| Group number | Age/sex group | Number of patients |
| --- | --- | --- |
| 1.1 | 0–5 years (male) |  |
| 1.2 | 0–5 years (female) |  |
| 2.1 | 6–17 years (male) |  |
| 2.2 | 6–17 years (female) |  |
| 3.1 | 18–39 years (male) |  |
| 3.2 | 18–39 years (female) |  |
| 4.1 | 40–64 years (male) |  |
| 4.2 | 40–64 years (female) |  |
| 5.1 | over 65 years (male) |  |
| 5.2 | over 65 years (female) |  |

2.3.2 Please, specify the approximate percentage change for patients' visits by the following age and sex groups for a period from 24 February 2022 to 1 December 2022 in comparison to the pre-war period:

| Group number | Age/sex group | Approximate change, %  (for example 20% increase or -10% decrease) |
| --- | --- | --- |
| 1.1 | 0–5 years (male) |  |
| 1.2 | 0–5 years (female) |  |
| 2.1 | 6–17 years (male) |  |
| 2.2 | 6–17 years (female) |  |
| 3.1 | 18–39 years (male) |  |
| 3.2 | 18–39 years (female) |  |
| 4.1 | 40–64 years (male) |  |
| 4.2 | 40–64 years (female) |  |
| 5.1 | over 65 years (male) |  |
| 5.2 | over 65 years (female) |  |

2.4 Did the number of veterans (in particular soldiers, officers, volunteers in connection with the military aggression of the Russian Federation against Ukraine which began on 24 February 2022; ex-combatants of the anti-terrorist operation/out of service) among patients change significantly since 24 February 2022?

1. Yes, increased (please, specify the approximate percentage change)
2. Yes, decreased (please, specify the approximate percentage change)
3. No
4. Not sure

   2.5.1 Which health conditions among current patients who are seeking medical care have increased?

   For example, the number of patients with mental health conditions has increased since 24 February 2022.

   *Open question*

   2.5.2 Which health conditions among current patients who are seeking medical care have decreased or have not changed?

   *Open question*

   2.5.3 Do health conditions among veterans who are seeking medical care differ from the other patients?
5. Yes/please specify the list of major health conditions of veterans seeking medical care
6. No
7. Not sure

   2.6 Has the main reason for contacting the PHC facility changed?
8. Yes
9. No
10. Not sure

    If the responder answers Yes to Question 2.6, please ask the following question.

    2.6.1 Please, specify the changes in the reasons for contacting the PHC facility.

    For example, more patients with children contact the PHC facility to vaccinate children within routine vaccination or less patients with suspected or with COVID-19 contact the PHC facility to perform the testing or less patients contact the PHC facility to do vaccination for COVID-19.

    *Open question*


    **Service delivery**

    3.1 Did the proportion of the following types of interactions between the health care worker and the patient change since 24 February 2022?

| Type of patients | Type of interaction | Yes, increased | Yes, decreased | No change | Not sure |
| --- | --- | --- | --- | --- | --- |
| Patients with declarations | In the PHC facility |  |  |  |  |
|  | Home visit |  |  |  |  |
|  | By phone |  |  |  |  |
|  | Via a video call/chat in messenger |  |  |  |  |
|  | Other (please specify) |  |  |  |  |
| Patients without declarations | In the PHC facility |  |  |  |  |
|  | Home visit |  |  |  |  |
|  | By phone |  |  |  |  |
|  | Via a video call/chat in messenger |  |  |  |  |
|  | Other (please specify) |  |  |  |  |

Responders should tick the right block for each type of interaction.

If the responder answers 'Yes, increased' or 'Yes, decreased' on any of the interactions, please ask the following question on each type of interaction.

3.1.1 Which health care worker does this interaction (in the PHC facility, home visit, …) most often occur with?

1. Family doctor
2. Nurse
3. Nonmedical staff
4. Not sure

   3.2 How did the main patient needs for medical care change since the beginning of the war at the PHC facility?

| Patient need for medical care | Increased | Decreased | No change | Not sure |
| --- | --- | --- | --- | --- |
| Visiting the family doctor |  |  |  |  |
| Need to renew prescriptions |  |  |  |  |
| Need for a referral to a specialist at the primary level |  |  |  |  |
| Need for a referral to a specialist at the secondary level |  |  |  |  |
| Visiting a specialist at the primary level |  |  |  |  |
| Laboratory testing |  |  |  |  |
| Diagnostics using medical equipment (CT, X-ray etc) |  |  |  |  |
| Vaccination within national vaccination schedule |  |  |  |  |
| Vaccination for COVID-19 |  |  |  |  |
| Supportive therapy (injections, IVs etc) |  |  |  |  |

3.2.1 If there have been changes in patient needs that are not listed in the previous question since the start of the full-scale invasion at the facility where you work, please indicate what those needs are and how they have changed (needs increased or decreased).

*Open question*

3.3 Did the staff of your facility have to change the number of working hours since 24 February 2022 due to changes in patient needs?

| Patient need for medical care | Yes, duration of working time increased due to increase of the workload | Yes, duration of working time decreased due to decrease of the workload | No change | Not sure |
| --- | --- | --- | --- | --- |
| Visiting the family doctor |  |  |  |  |
| Need to renew prescriptions |  |  |  |  |
| Need for a referral to a specialist at the primary level |  |  |  |  |
| Need for a referral to a specialist at the secondary level |  |  |  |  |
| Visiting a specialist at the primary level |  |  |  |  |
| Laboratory testing |  |  |  |  |
| Diagnostics using medical equipment (CT, X-ray etc) |  |  |  |  |
| Vaccination within national vaccination schedule |  |  |  |  |
| Vaccination for COVID-19 |  |  |  |  |
| Supportive therapy (injections, IVs etc) |  |  |  |  |

3.3.1 If in question 3.2.1 you indicated that since the beginning of the full-scale invasion in the facility where you work, there have been changes in other patient needs, please specify whether the staff of your facility had to change the work schedule from Feb 24, 2022, due to the change in needs (to increase or decrease the duration of working hours).

3.4 Do you receive any assistance from institutions/organizations that provide social care/community-based care in your region?

1. Yes/If yes, please specify the organizations and the services they deliver
2. No
3. Not sure

   3.5 In your opinion, has the patient pathway changed since 24 February 2022?
4. Yes
5. No
6. Not sure

   If the responder answers Yes to 3.5, please ask the following question.

   3.5.1 In your opinion, did the changes occur on the following stages of the patient pathway?

| Stages of the patient pathway | Yes | No | Not sure |
| --- | --- | --- | --- |
| Appointment |  |  |  |
| Visit |  |  |  |
| Specialist referral |  |  |  |
| Diagnostics |  |  |  |
| Treatment prescription |  |  |  |
| Follow-up |  |  |  |

Responders should tick the right block for each stage of the patient pathway.

If the responder ticks the block Yes on any of the rows, please, answer the following question for each such row.

3.5.2 Please specify the changes on the following stages of the patient pathway.

Open question

3.5.3 Did the changes occur on the other stages of the patient pathway that were not previously mentioned? If so, please specify the changes on these stages of the patient pathway.

Open question

**DISRUPTIONS AFFECTING THE WORK OF THE PHC FACILITY**

4.1 Since February 24th, 2022, on a scale from 1 to 5, to which extent was your PHC facility affected by the following disruptions, where:

5 = being heavily affected in a way that it was not possible to serve patients,

4 = heavily affected but some services remained,

3 = affected, but majority of services remained,

2 = affected only some services,

1 = did not affect at all.

| No. | List of disruptions | 5 | 4 | 3 | 2 | 1 | Not sure |
| --- | --- | --- | --- | --- | --- | --- | --- |
| 1.1 | Medical workforce shortages – physicians |  |  |  |  |  |  |
| 1.2 | Medical workforce shortages – nurses |  |  |  |  |  |  |
| 1.3 | Nonmedical workforce shortages |  |  |  |  |  |  |
| 2 | Increased number of patients (e.g. IDPs) |  |  |  |  |  |  |
| 3 | Damage to health facilities |  |  |  |  |  |  |
| 4 | Disruptions to laboratory testing |  |  |  |  |  |  |
| 5 | Supply chain disruption for medical equipment, supplies etc. |  |  |  |  |  |  |
| 6 | Supply chain disruption for medicines |  |  |  |  |  |  |
| 7 | Depreciation of UAH/Increase of prices |  |  |  |  |  |  |
| 8 | Deteriorated mental health of the facility workers |  |  |  |  |  |  |
| 9 | Water supply cuts |  |  |  |  |  |  |
| 10 | Electricity cuts |  |  |  |  |  |  |
| 11 | Heat supply cuts |  |  |  |  |  |  |

Responders should tick the right block for each disruption.

4.2 Did the work of your PHC facility experience any other disruptions?

1. Yes/Please specify
2. No
3. Not sure

   If the responder puts 2 to 5 in Q 4.1, please ask the following question on each disruption mentioned by the responder

   4.3 In your opinion, which cost categories/procurements/investments and how were affected by the disruption?

| №No. | Disruption/challenge specified by the responder in Q 4.1 | Yes, increased the cost | Yes, decreased the cost | No | Not sure |
| --- | --- | --- | --- | --- | --- |
| 1.1 | Salary (medical workforce) |  |  |  |  |
| 1.2 | Salary (nonmedical workforce) |  |  |  |  |
| 2 | Medicines and immunobiological drugs |  |  |  |  |
| 3 | Medical devices |  |  |  |  |
| 4 | Disinfectants |  |  |  |  |
| 5 | Personal protective equipment |  |  |  |  |
| 6 | Diagnostic tests (tests performed directly by the provider and outsourced (external service) |  |  |  |  |
| 7 | Stationery (including forms, special accounting journals, sign boards, signs, plates, printing products, periodicals, etc.) |  |  |  |  |
| 8 | Household materials, inventory |  |  |  |  |
| 9 | Fuel and lubricating materials |  |  |  |  |
| 10 | Utilities |  |  |  |  |
| 11 | Repair (current) |  |  |  |  |
| 12 | Building materials |  |  |  |  |
| 13 | Other operating costs |  |  |  |  |
| 14 | Reimbursement of the cost of discounted drugs, insulins |  |  |  |  |
| 15 | Capital investments |  |  |  |  |
| 16 | Purchase of goods and material values |  |  |  |  |

Responders should tick the right block for each cost category /purchase/ investment.

If the responder ticks "Yes, increased" or "Yes, decreased" on any of the cost categories, ask the following question on each cost category /purchase/investment where the changes occurred.

4.3.1 Please, indicate the approximate percentage change in the specified cost/purchase/investment.

| Cost/purchase/investment | Approximate change, %  (for example 20% increase or -10% decrease) |
| --- | --- |
| Medical devices |  |

***Sources of funding***

5.1 Did the funding received from the NHSU through capitation payments change since 24 February 2022?

1. Yes, it increased
2. Yes, it decreased
3. No, it did not change
4. Not sure

   5.2 Did other sources of funding change since 24 February 2022?

|  | Sources of funding | Yes, increased | Yes, decreased | No | Not sure |
| --- | --- | --- | --- | --- | --- |
| 1 | Humanitarian and charitable aid |  |  |  |  |
| 2 | Funding from local authorities |  |  |  |  |
| 3 | Additional funding from national budget |  |  |  |  |
| 4 | Paid services |  |  |  |  |
| 5 | Other/please specify |  |  |  |  |

Responders should tick the right block for each source of funding.

If the responder indicates the funding change of any of the sources in Q 5.2, please ask the following question on each source mentioned by the responder.

5.2.1 Please indicate which cost categories are covered by the mentioned funding sources. Please, put the percentage share of each funding source, 100% in total per each cost category.

| № | Covered cost categories, 100% in total | NHSU Funding, % | Humanitarian and charitable aid, % | Funding from local authorities, % | Additional funding from national budget, % | Paid services, % | Other sources) |
| --- | --- | --- | --- | --- | --- | --- | --- |
| 1.1 | Salary (medical workforce) |  |  |  |  |  |  |
| 1.2 | Salary (nonmedical workforce) |  |  |  |  |  |  |
| 2 | Medicines and immunobiological drugs |  |  |  |  |  |  |
| 3 | Medical devices |  |  |  |  |  |  |
| 4 | Disinfectants |  |  |  |  |  |  |
| 5 | Personal protective equipment |  |  |  |  |  |  |
| 6 | Diagnostic tests (tests performed directly by the provider and outsourced (external service) |  |  |  |  |  |  |
| 7 | Stationery (including forms, special accounting journals, sign boards, signs, plates, printing products, periodicals, etc) |  |  |  |  |  |  |
| 8 | Household materials, inventory |  |  |  |  |  |  |
| 9 | Fuel and lubricating materials |  |  |  |  |  |  |
| 10 | Utilities |  |  |  |  |  |  |
| 11 | Repair (current) |  |  |  |  |  |  |
| 12 | Building materials |  |  |  |  |  |  |
| 13 | Other operating costs |  |  |  |  |  |  |
| 14 | Reimbursement of the cost of discounted drugs, insulin |  |  |  |  |  |  |
| 15 | Capital investments |  |  |  |  |  |  |
| 16 | Purchase of goods and material values |  |  |  |  |  |  |

Responders should put the percentage share of each funding source, 100% in total per each cost category.

***PROVIDER NEEDS***

6.1 What are the current needs of your PHC facility?

For example, purchase of autonomous power supply devices, purchase of medical drugs for stock, staffing needs for patients with mental health conditions etc.

*Open question*

6.2 Which of the mentioned needs come up after 24 February 2022?

*Open question*

Appendix 2. Interview guide

| Question | Comment |
| --- | --- |
| **General part** | |
| How did the full-scale invasion affect the work of your facility? | Consider it as a logical start of the interview. Please help the interviewee to mention the indirect and direct impact on 3 main topics of the interview - patient profile, service delivery & need for services, revenues & expenditures |
| Were there any other reasons that impacted the work of your facility since February 24, 2022 (except a full-scale invasion)? | The interviewee can use this information further during the interview when talking about the changes in the work of the facility. Possible topics: NHSU regulations, changes in e-Health system, logistic chains, etc. |
| **Patient profile** | |
| Please describe changes in the patient profile following the beginning of the war? Please consider patient age and gender structure, IDPs, veterans etc.?  Have these changes occurred directly or not directly as a result of the war or have there been any other reasons impacting the patient profile? Please describe. | Check whether changes impact patients in the patient list or serve also patients outside their own patient lists. |
| Please describe whether and how have the changes in the patient profile affected the work of your facility?  Did you have to make any changes in the work of the facility due to the changes in the health conditions? If yes, please describe these changes and specific health conditions. | If changes in health conditions are mentioned |
| Despite this, did you notice any change in the reasons for contacting the facility among current patients? | Please keep in mind:   - Vaccination according to national calendar - Medical certificates - COVID-19 - Mental health problems - Regular checks   Check whether changes impact patients in the patient list or serve also patients outside their own patient lists. |
|  |  |
| **Changes in service delivery** | |
| Have you made any changes in operational processes of your facility due to the war, if yes, please describe? E.g. have you needed to hire or fire staff, extend opening hours, provide more remote consultations, simplify the process for prescribing or dispensing medicines etc. | Please help interviewees to consider the direct and indirect impact of war.  If the provider describes changes in operational processes, please specify what may have been the specific cause for each change described.  If interviewee does not feel that there is no impact, ask whether they in general have made any changes |
| Please think of different services provided in your practice (diagnostics, referrals, prescriptions, vaccinations, paid services etc). Please describe, have there been significant changes in the need for different services and which kind. What may be the specific cause of it? | Please motivate interviewees to think about the different service types.  Keep in mind:  Visiting the family doctor  Need to renew prescriptions  Need for a referral  Laboratory testing  Diagnostics  Vaccination  Supportive therapy  Paid services  If changes are identified, please elaborate which kind of patients have initiated the change and why. |
| Please describe a usual patient pathway during seeking care in the healthcare system. Please elaborate whether and how this may have changed because of the war. | You may provide an example on a patient pathway.  Please specify whether there have been changes in data exchanges with other providers, referral process to secondary care.  Keep in mind:  Appointment  Visit  Specialist referral  Diagnostics  Treatment prescription  Follow-up |
| Have there been any disruptions caused by the war that have affected the work of the facility, please describe how and how often these disruptions have happened e.g. electricity cuts, water supply cuts, heat supply cuts, medical and non-medical workforce shortages, deteriorated mental health of the facility workforce, supply chain disruptions, damage to health facilities, disruptions to laboratory testing etc. Please describe how you have solved these disruptions. Please describe any mitigation measures to avoid disruptions in the future. | Please motivate interviewee to discuss every disruption, how long it took place, how it impacted service delivery and access to services, how it was solved. |
| **Changes in revenues and expenditures** | |
| Considering the above, please describe changes to the facility expenditures which are directly related to changes in patient profile and service needs. | Please ask the interviewee to think of different expenditure categories e.g. salaries, equipment etc and describe the scale of impact etc. |
| Please describe any other changes in the expenditures that may have happened as a result of the war e.g. inflation, increase in fuel prices etc | Please ask the interviewee to describe the scale of impact and provide examples. |
| Please describe whether these cost increases have made you prioritize spending and cut expenses for any other activity, made you change service delivery practices e.g. postponing renewal of medical equipment, reducing spending on diagnostics etc. | Please also ask if they are able to meet the expenses and how, what happens if funds are insufficient. Please elaborate what cost they may have cut as a first priority to handle the increased expenses. |
| Please describe whether and what changes have there been for your revenues. What has impacted the change? Has there been change in revenues in NHSU provided funding, municipality provided funding, foreign aid, paid services etc.  What was your contribution in receiving additional funding from local authorities, donors etc.? | Please elaborate on whether there has been in kind or financial support. If there has been an increase or decline in revenues for paid services, please elaborate what may be the underlying cause for this. |
| Please describe whether and how did the war affect investment or procurement plans of the facility? | Focus on infrastructure and equipment planning. |
|  |  |
| **Additional questions** | |
| What did you have to change in service delivery processes due to the increase of patients without declarations’ visits? |  |
| How did you have to change the work of your facility due to the changes in the number of visits of veterans?  How did you have to change the processes due to the increase in need of medical certificates (abroad)? |  |
| Please, specify the changes in the process of renewing prescriptions since February 24, 2022. Are there any specific problems with renewing prescriptions? | Need to renew prescriptions - Increase |
| What are the reasons to make a referral to a specialist at the primary level since February 24, 2022? | Need for a referral to a specialist at the primary level - increase (13) |
| What did you have to change due to changes in the need for vaccination within the national vaccination schedule? Were there any changes / delays in the logistics’ processes of the vaccine? Did you experience a lack of any vaccines? | Vaccines  Logistics  Any delays |
| What are the main reasons to seek assistance from institutions/organizations that provide social care/community-based care in your region?  Did their assistance have a significant impact on the processes of your facility? | Social care |
| What are the main cost categories that were affected by the overall increase of prices? Did you have to contact any stakeholders to receive additional funding to cover costs (local authorities, ITA, NGOs)? | Disruption - increase of prices |
| What did you change in the operational processes of your facility due to electricity cuts? What are the changes in the cost spendings / investments? | Disruption - electricity cuts |
| Did revenues received from the NHSU change since February 24, 2022? If yes, why did this happen? | Increase / decrease of medical staff  Increase of declarations  Technical issues  Changes in regulations |
| What were the main cost categories covered by humanitarian and charitable aid since February 24, 2022? | Increase - humanitarian and charitable aid |
| What were the main cost categories covered by the funding from local authorities? | Funding from local authorities |
| What were the main categories covered by the funding from the national budget? | Funding from national budget |
| What are the changes in paid services? | Paid services |
| Is there any possibility to cover current needs of your PHC facility at the expense of the facility budget? |  |

Appendix 3: Informed consent form

**Impact of the conflict on the costs of primary health care and investments in Ukraine: survey of war effects**

**INTERVIEW CONSENT FORM**

The National Health Service of Ukraine, with the technical support of the World Health Organization, is conducting a study to analyze the impact of the war on the costs, procurement, and capital investments of primary health care (PHC) facilities. The study consists of several work streams: 1. Cost data collection to inform NHSU on revision of the capitation payments for PHC providers; 2. Qualitative research to complement the analysis with information about the impact of the war on provider service delivery models and expenditures; 3. Research on provider-level task distribution. To confirm our hypothesis based on the data collected from providers, we are conducting interviews with selected candidates. The criteria for selecting PHC facilities aligns with the criteria for the selection of PHC reference providers of the cost study^[[1]](#footnote-1)^. Your practice has been selected as one interviewee to provide us with valuable additional information. The study findings will be used to inform the policy dialogue offering valuable insights into the evolving PHC landscape in Ukraine during the invasion and possibilities for its future after the invasion, revealing the challenges faced and adaptations made in this challenging environment.

We would welcome this interview to be in a free format and not necessarily a question-answer type. We have some guiding questions, but your opinion and insights are prioritized. The interview will last about 1 to 1,5 hours. We would like to address the following topics:

- Changes in the patient profile
- Changes in service delivery, need for services
- Changes in revenues and expenditures.

We don’t anticipate any risks associated with your participation, but you have the right to stop the interview or withdraw from the research at any time. Ethical procedures for academic research require that interviewees explicitly agree to being interviewed and how the information contained in their interview will be used. This consent form is necessary for us to ensure that you understand the purpose of your involvement and that you agree to the conditions of your participation. Would you, therefore sign this form to certify that you approve the following:

| **Participant consent** | **Please circle as appropriate** |
| --- | --- |
| I confirm that I have read the information above about the study. | Yes / No |
| I confirm that I have had the opportunity to consider the information, ask questions and have had these answered satisfactorily. | Yes / No |
| I understand and agree that the recording will be used for any possible anonymous citations, and the discussion with me will be strictly confidential; all the information provided by me will be used in an anonymous form. | Yes / No |
| I give permission for you to use quotations from the interview without using information that could identify me or the name of the organization I work for. | Yes / No |
| I understand that I don’t have to talk about things that I don’t want to talk about. I know that I can stop our interview at any time and without giving a reason. | Yes / No |
| I agree for the interview to be recorded and a transcript to be produced. | Yes / No |
| I agree that the transcript of the interview will be analysed by the research investigator and the research team with whom he might collaborate as part of the research process. | Yes / No |
| I agree for the data I provide to be archived. I understand that other authenticated researchers will have access to this data only if they agree to preserve the confidentiality of the information as requested in this form. I understand that other authenticated researchers may use my words in publications, reports, web pages, and other research outputs, only if they agree to preserve the confidentiality of the information as requested in this form. | Yes / No |

Name of participant: __________________________

Signature of participant: __________________________

Date (DD/MM/YYYY): __________________________

Thank you for consenting to be involved in our study. If you have any questions about the study, please contact Yaroslav Kudlatskyi, LLC “CIVITTA UKRAINE” at mobile phone: +380937653679 or email: yaroslav.kudlatskiy@civitta.com.

*(неофіційний переклад)*

**Вплив конфлікту на витрати на первинну медичну допомогу та інвестиції в Україні: дослідження наслідків війни**

**ФОРМА ЗГОДИ НА ІНТЕРВ'Ю**

Національна служба здоров'я України за технічної підтримки Всесвітньої організації охорони здоров'я проводить дослідження з метою аналізу впливу війни на витрати, закупівлі та капітальні інвестиції закладів первинної медичної допомоги (ПМД). Дослідження складається з кількох блоків: 1) збір даних про витрати для інформування НСЗУ щодо перегляду капітаційних виплат для надавачів ПМД; 2) якісне дослідження для доповнення аналізу інформацією про вплив війни на моделі надання послуг та витрати надавачів; 3) дослідження розподілу завдань на рівні надавачів. Для підтвердження наших гіпотез на основі даних, отриманих від надавачів, ми проводимо інтерв'ю з відібраними кандидатами. Критерії відбору закладів ПМД збігаються з критеріями відбору референтних надавачів ПМД в рамках дослідження про витрати^[[2]](#footnote-2)^. Ваш заклад було обрано в якості одного з респондентів для проведення інтерв'ю, щоб надати нам цінну додаткову інформацію. Результати дослідження будуть використані для інформування політичного діалогу, пропонуючи цінну інформацію про розвиток ПМД в Україні під час вторгнення та можливості для її майбутнього після вторгнення, розкриваючи проблеми, з якими зіткнулися надавачі, та адаптацію, здійснену в цьому складному середовищі.

Ми будемо раді, якщо це інтерв'ю буде у вільному форматі і не обов'язково у вигляді запитань-відповідей. У нас є кілька примірних запитань, але Ваша думка та інсайти є пріоритетними. Інтерв'ю триватиме від 1 до 1,5 годин. Ми хотіли б обговорити наступні теми:

- Зміни в профілі пацієнта
- Зміни в наданні послуг, потреба в послугах
- Зміни в доходах і витратах.

Ми не передбачаємо жодних ризиків, пов'язаних з Вашою участю, але ви маєте право зупинити інтерв'ю або вийти з дослідження в будь-який час. Етичні процедури академічних досліджень вимагають, щоб респонденти давали пряму згоду на проведення інтерв'ю і на те, як буде використовуватися інформація, що міститься в інтерв'ю. Ця форма згоди необхідна нам для того, щоб переконатися, що Ви розумієте мету Вашої участі та погоджуєтеся з умовами Вашої участі. Тому, будь ласка, підпишіть цю форму, щоб засвідчити, що Ви погоджуєтеся з наступним:

| **Згода учасника** | **Будь ласка, обведіть відповідне у коло** |
| --- | --- |
| Я підтверджую, що ознайомився/ознайомилась з наведеною вище інформацією про дослідження. | Так / Ні |
| Я підтверджую, що мав/мала можливість ознайомитися з інформацією, поставити запитання та отримати на них задовільні відповіді. | Так / Ні |
| Я розумію і погоджуюся, що запис буде використаний для будь-яких можливих анонімних посилань, а обговорення зі мною буде суворо конфіденційним; вся надана мною інформація буде використана в анонімній формі. | Так / Ні |
| Я даю дозвіл на використання цитат з інтерв'ю без використання інформації, яка може ідентифікувати мене або назву закладу, в якому я працюю. | Так / Ні |
| Я розумію, що не зобов'язаний/зобов'язана говорити про те, про що не хочу говорити. Я знаю, що можу припинити інтерв'ю в будь-який момент і без пояснення причин. | Так / Ні |
| Я даю згоду на те, щоб інтерв'ю було записано та складено його стенограму. | Так / Ні |
| Я погоджуюся, що стенограма інтерв'ю буде проаналізована дослідником та дослідницькою командою, з якою він може співпрацювати в рамках дослідницького процесу. | Так / Ні |
| Я погоджуюся на те, що надані мною дані будуть архівуватися. Я розумію, що інші авторизовані дослідники матимуть доступ до цих даних лише за умови, що вони погодяться зберігати конфіденційність інформації відповідно до запиту в цій формі. Я розумію, що інші авторизовані дослідники можуть використовувати мої слова в публікаціях, звітах, веб-сторінках та інших результатах досліджень, тільки якщо вони погоджуються зберігати конфіденційність інформації, як зазначено в цій формі. | Так / Ні |

Ім'я учасника: __________________________

Підпис учасника __________________________

Дата (ДД/ММ/РР): __________________________

Дякуємо, що погодилися взяти участь у нашому дослідженні. Якщо у Вас виникли запитання щодо дослідження, будь ласка, звертайтеся до Ярослава Кудлацького, ТОВ «СІВІТТА УКРАЇНА», моб: +380937653679 або електронною поштою: yaroslav.kudlatskiy@civitta.com.

1. Results of costing for provider payments in primary health care in Ukraine: technical report. Copenhagen: WHO Regional Office for Europe; 2024. Licence: CC BY-NC-SA 3.0 IGO ([Results of costing for provider payments in primary health care in Ukraine: technical report](https://www.who.int/europe/publications/i/item/WHO-EURO-2024-8277-48049-71211)). [↑](#footnote-ref-1)
2. Результати розрахунку витрат на оплату послуг надавачів первинної медичної допомоги в Україні: технічний звіт. Копенгаген: Європейське регіональне бюро ВООЗ; 2024. Ліцензія: CC BY-NC-SA 3.0 IGO ([Результати розрахунку витрат на оплату послуг надавачів первинної медичної допомоги в Україні: технічний звіт](https://www.who.int/europe/publications/i/item/WHO-EURO-2024-8277-48049-71211)). [↑](#footnote-ref-2)
